# Distribution of Toxigenic *Halomicronema* spp. in Adjacent Environments on the Island of Ischia: Comparison of Strains from Thermal Waters and Free Living in *Posidonia Oceanica* Meadows

**DOI:** 10.3390/toxins11020099

**Published:** 2019-02-08

**Authors:** Valerio Zupo, Mirko Mutalipassi, Nadia Ruocco, Francesca Glaviano, Antonino Pollio, Antonio Luca Langellotti, Giovanna Romano, Maria Costantini

**Affiliations:** 1 Department of Marine Biotechnology, Stazione Zoologica Anton Dohrn, 80121 Naples, Italy; mirko.mutalipassi@szn.it (M.M.); nadia.ruocco@szn.it (N.R.); francesca.glaviano@szn.it (F.G.); romano@szn.it (G.R.); mcosta@szn.it (M.C.); 2 Department of Biology, University of Naples Federico II, Complesso Universitario di Monte Sant’Angelo, Via Cinthia, 80126 Naples, Italy; anpollio@unina.it; 3 CAISIAL, Aquaculture Division, University of Naples Federico II. Via Università, 80055 Portici (NA), Italy; langello@unina.it

**Keywords:** toxicology, thermal water, marine, adaptation, evolution, spent medium

## Abstract

Organisms adaptable to extreme conditions share the ability to establish protective biofilms or secrete defence toxins. The extracellular substances that are secreted may contain monosaccharides and other toxic compounds, but environmental conditions influence biofilm characteristics. Microorganisms that are present in the same environment achieve similar compositions, regardless of their phylogenetic relationships. Alternatively, cyanobacteria phylogenetically related may live in different environments, but we ignore if their physiological answers may be similar. To test this hypothesis, two strains of cyanobacteria that were both ascribed to the genus *Halomicronema* were isolated. *H. metazoicum* was isolated in marine waters off the island of Ischia (Bay of Naples, Italy), free living on leaves of *Posidonia oceanica*. *Halomicronema* sp. was isolated in adjacent thermal waters. Thus, two congeneric species adapted to different environments but diffused in the same area were polyphasically characterized by microscopy, molecular, and toxicity analyses. A variable pattern of toxicity was exhibited, in accordance with the constraints imposed by the host environments. Cyanobacteria adapted to extreme environments of thermal waters face a few competitors and exhibit a low toxicity; in contrast, congeneric strains that have adapted to stable and complex environments as seagrass meadows compete with several organisms for space and resources, and they produce toxic compounds that are constitutively secreted in the surrounding waters.

## 1. Introduction

Filamentous cyanobacteria are ubiquitous and widely distributed across many environments, including freshwater, seawater, and terrestrial habitats [1]; they are present in the benthos, in the water column, and even in the body of specific hosts [2]. Proliferations of benthic mat-forming cyanobacteria belonging to *LPP* groups have been reported in various sites in the world, and they commonly produce a range of neurotoxins that are collectively known as anatoxins, which pose risks to human and animal health. Due to their overall similarity, they are frequently indistinguishable from a morphological point of view, and recently, Komarek et al. [3] suggested that when possible, it should be preferable to circumscribe smaller monophyletic genera, avoiding polyphyletic genera including not related species [4]. Often, they dominate extreme environments, as they are protected by a film of extracellular polymeric substances (EPS) that makes them adapted to the lowest (Antarctic [5]) or highest (thermal environments [6]) temperatures and salinities, as well as low irradiances [7]. Many cyanobacteria produce toxins that, according to their effects, can be pooled into five groups: cytotoxins, dermatotoxins, hepatotoxins, neurotoxins, and irritant toxins [8]. Cyanobacteria can shape the dynamics of aquatic microbial communities [9] due to the production of cyanotoxins (a diverse group of compounds, both from the chemical and the toxicological points of view) as depsipeptides, portoamides, and halogenated organic compounds [10,11] that exhibit clear toxigenic effects toward other bacteria, eukaryotic algae, protozoans, and various invertebrates [12], dramatically impacting the diversity of aquatic communities [13]. Furthermore, cyanobacteria-dominated blooms, which are characterized by marked seasonal patterns, have been observed in several freshwater basins [14]. In addition, cyanobacteria are known to produce homoanatoxin-a, microcystins, and saxitoxins [15,16,17,18]. Saxitoxins trigger the blockage of sodium ion channels and the inhibition of impulse generation in peripheral nerves and skeletal muscles. Anatoxins (acetylcholinesterase inhibitors) may mimic acetylcholine functions, since they bind to muscle acetylcholine receptors, inducing contraction. These toxins continuously stimulate the muscles until paralysis occurs [19]. Hepatotoxins, in their turn, may be divided in three groups (microcystins, nodularins, and cylindrospermopsins), according to their chemical nature. Microcystins are probably the prevalent cyanotoxins in the environment, and they are the most studied group among cyanobacteria bioactive compounds [20]. They form a family of monocyclic heptapeptides containing the unusual β-amino acid Adda (3-amino-9-methoxy-2,6,8-trimethyl-10-phenyldeca-4E, 6E-dienoic acid) and represent possible hazards for human beings when dissolved in drinking water [21]. On the whole, they may produce sub-chronic and chronic toxicity along with tumor promotion. Nodularins have a structure similar to that of microcystins except for the number of amino acids, while cylindrospermopsins are alkaloids produced by *Cylindrospermopsis* spp., *Umezakia* spp., and *Aphanizomenon* spp. [12], producing pathological changes in the liver, kidneys, spleen, thymus, and heart of mammals, birds, and fish [22,23]. However, other compounds that are responsible for these effects belong to various classes of chemicals, ranging from alkaloids to aromatic compounds, cyclophanes, fatty acids, macrolides, nucleoside, peptides, polyketides, and terpenoids [24]. Since their presence may produce acute toxicity for animals and humans [17], and their natural blooms correspond to deadly conditions for various organisms that are present in the same communities [25], there is rising awareness of the risk that thin filamentous cyanobacteria proliferations pose to human and animal health [26,27]. The list of cyanobacteria secondary metabolites elongates continuously, and they embody important, still underexplored organisms for the discovery of new drugs, cosmeceuticals, and anti-inflammatory drugs [28]. Cyanobacteria also produce volatile compounds such as geosmin, whose toxicity was demonstrated [29]. In parallel, bloom-associated cyanobacteria produce toxins impacting their ecosystems and the surroundings, bringing the associated communities to hypoxia. 

The ecological role of toxin produced by cyanobacteria is still debated: apart from being produced to discourage predation, they probably play multiple functions in cellular metabolism, in particular counteracting oxidative stresses, and facilitating the uptake of nutrients, particularly iron [30]. However, the microbial community itself may mitigate their effects under some ecological conditions, since the allelopathic behavior and the production of secondary metabolites may be influenced by the nutrient availability and other interactions with the resident microbiota, as well as by abiotic factors such as salinity and irradiance [31]. Several uncertainties persist regarding the environmental triggers that facilitate the formation of cyanobacteria blooms and their persistence [32], but it has been demonstrated that various abiotic factors, including nutrient loads, salinity, and temperature, influence the production of toxins and their release in the surrounding environments [33]. From this perspective, it is likely that the increased frequency of toxic blooms [32] that has been observed in the last decades may be an indirect result of global warming [34,35]. The consequences of global climate changes (elevated temperature, increased atmospheric concentrations of carbon dioxide, elevated ultraviolet (UV) fluxes) also depend on cyanobacterial ecology and growth [36]. Eutrophication and higher temperatures trigger a higher release of cyanotoxins in waters, increasing the ecological risk [37,38]. Trends emerging from previous research [28] indicate that the functions of these microbes within a given environment may be highly conserved, going beyond their phylogenetic relationships and taxonomic identities [39]. Thus, it is worth investigating the relationships among taxonomically-related strains of cyanobacteria and their specific toxicity, especially when their populations are established in extreme environments [40,41]. 

We have recently isolated two strains of cyanobacteria, which are both ascribed to the genus *Halomicronema,* and have both been established as new species in the last decades [42,43], but are linked to very different environments. The first strain belongs to *Halomicronema metazoicum (Caroppo, Pagliara, Albertano, 2012)*. It was previously known to be an endobiont of marine metazoans [42], and it has only recently [44] been found to be also associated with the epiphytic community of a *Posidonia oceanica* (L.) Delile meadow [45] off the island of Ischia. The second strain was attributed to *Halomicronema* sp. It is considered to be “a benthic, moderately halophilic and thermophilic genus of cyanobacteria with very thin trichomes” [43], and it has been isolated in thermal waters on the island of Ischia. Thus, both species were present in the same area: the first in a typically stable seawater environment, and the second in thermal waters. Their morphology is quite similar, since they both appear to be non-heterocystous, thin filamentous oxygenic prototroph cyanobacteria exhibiting trichomes that have a diameter of less than two μm [46]. In addition, the first was previously known only as an endobiont of metazoans (and it was found, in contrast, free living as an epiphyte), while the second was considered “moderately thermotolerant”, but it was isolated in thermal waters at a higher temperature. In addition, the latter was considered to be “benthic”, since it was initially isolated from benthic microbial mats [43], but it has been demonstrated to produce blooms in the water column when high concentrations of nutrients are available. The availability of two related species of cyanobacteria living in such different environments permits investigating the factors influencing the interactions between microalgae and bacteria, and those determining the wide ecological success of the latter [47]. In fact, interactions within these organisms are still poorly understood and are affected by various environmental features as limiting resources and competition issues. For example, some cyanobacteria were reported to use toxins as a response to nutrient depletion and related allelopathic interactions, and these can contribute to their expansion, even when nutrient loading is limited [48]. The presence of two taxonomically related species in the same area will possibly explain the evolution and dispersion of cyanobacteria in different environments [4]. For these reasons, we present here a polyphasic analysis of two novel strains that have been recently isolated from very different environments in the same geographical area; the analysis includes their morphological description along with their ultrastructural, physiological, and molecular characterisation. Our aim is to test the hypothesis that the ability of cyanobacteria to produce toxic weapons has evolved according to environmental constraints, and that a stronger presence of possible competitors promotes the evolution of a higher toxigenic activity. 

## 2. Results

### 2.1. Halomicronema Metazoicum

**Figure 1:**
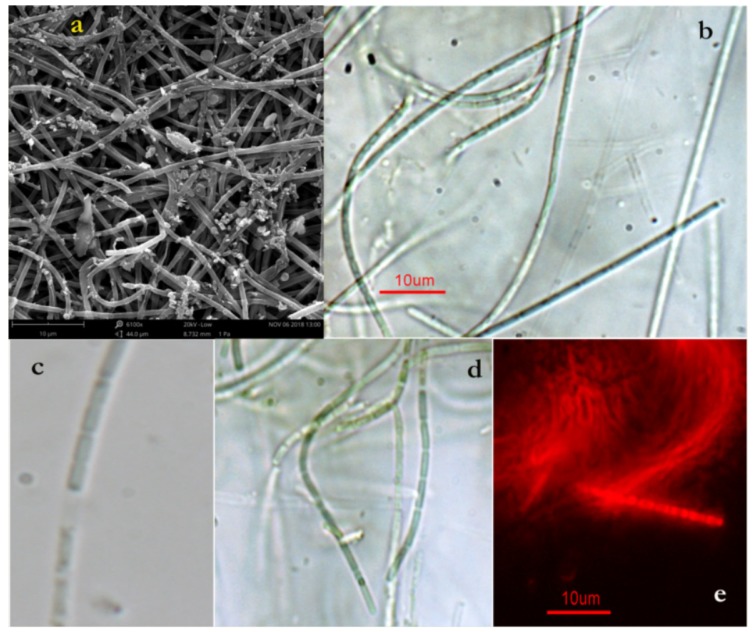
Photomicrographs of *H. metazoicum* mats grown in *f/2* medium. (**a**) Structure of the surface of a benthic mat observed at the high performance desktop SEM Phantom ProX (gentle concession of Alfatest, ThermoFisher Scientific, Rome, Italy), showing thin filaments and mucous secretions at 6100×; (**b**) occurrence of a papilla at the apex of a trichome at 100×; (**c**) necridic cell between two neighboring cells at 100×; (**d**) cells of each trichome are cylindrical, with constrictions at the cross walls at 100×; (**e**) light fluorescence micrograph of filaments surrounded by an amorphous colorless sheath at 100×.

The strain collected on *Posidonia oceanica* leaves in Lacco Ameno d’Ischia (Italy) and grown in *f/2* medium, according to our micromorphology analyses, exhibited filaments grouped into emerald-green macroscopic aggregates, which are generally not adherent to the culture vessel (Figure 1). Their sheath was colorless, generally thin, amorphous, and somewhat diffused around the trichome, but rarely extending over the trichome apex. Cells had a diameter of 0.8 to 1.0 μm, and they were cylindrical, elongated, and usually two to five μm long. Terminal cells were rounded, exhibiting constrictions at the cross wall. Reproduction was observed to occur by fragmentation in a few-celled hormogonia. The strains exhibited 99% pairwise sequence identity with *Halomicronema metazoicum* ITAC101 (isolated for the first time by Caroppo et al. [42]) according to the molecular characterization. The strain of cyanobacteria that was isolated from leaves of *Posidonia*, named *Cyano_Pos*, was phylogenetically well-separated from *H. excentricum,* with a 93.5% pairwise sequence identity.

### 2.2. Halomicronema sp.

**Figure 2:**
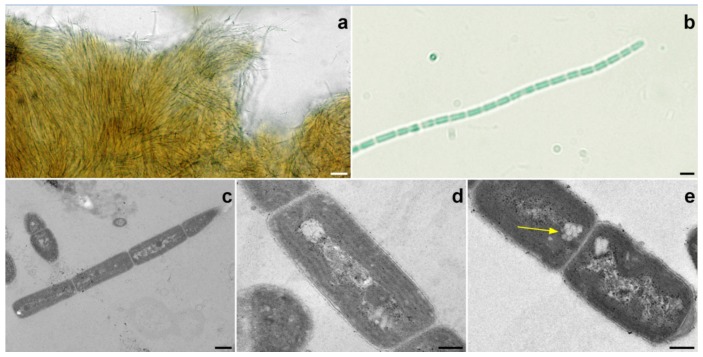
(**a**) Photomicrographs of *Halomicronema* sp. mats grown on agarized BBM medium at 20×. Bar scale = 20 μm; (**b**) photomicrograph of a *Halomicronema* sp. filament at 100×. Bar scale = 2 μm. Ultrastructure (TEM) of *Halomicronema* sp. (**c**) four-celled hormogonium, with a tapered apical cell at 8000×. Bar scale = 0.5 μm; (**d**) disposition of the thylakoids, arranged in four rows, parallel to the longitudinal axis of the cell at 20,000×. Bar scale = 0.2 μm; (**e**) gas vacuoles (arrow) apically placed at 20,000×. Bar scale = 0.2 μm.

The strain collected in thermal waters and grown on agarized BBM medium formed a brownish-green mat, with thin filaments grouped in layered fascicles (Figure 2a). In liquid cultures, filaments were pale-blue green, with a very thin colorless sheath, and straight or slightly bent, without false ramifications. Trichomes consisted of elongated cells with a length/diameter ratio higher than one; the apical cell appeared rounded, with a papilla (Figure 2b). Cell wall constrictions were not clearly visible under optical microscopy. Trichomes are divided in non-motile segments, without necridic cells. Transmission electron microscopy (TEM) revealed the presence of moderate constrictions at the cross walls (Figure 2c). Thylakoids are arranged concentrically to from three to five rows that run parallel to the longitudinal axis of the cell (Figure 2d). The multiplication of filaments takes place by fragmentation. The apical ends of hormogonia are long-pointed after trichome breakage, and had no calyptra. Gas vesicles are present (Figure 2e). 

**Figure 3:**
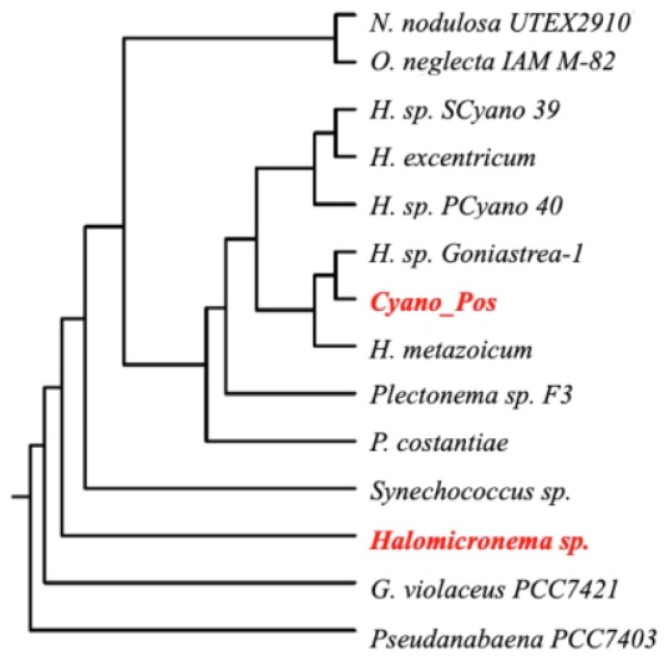
Phylogenetic tree based on 16S rRNA gene sequences. Strains analysed in this work, *Cyan_Pos*, and *Halomicronema* sp., are indicated in red.

16S rRNA gene analysis showed, as sequence-producing significant alignments, “uncultured Oscillatoriales cyanobacterium”. Phylogenetic analysis of this strain along with another 13 cyanobacteria (phylogenetically related [44]) revealed that it was well-separated from *Cyano Pos* with 89% sequence identity (Figure 3). Furthermore, this thermal water cyanobacterium exhibited about 90% pairwise sequence identity with *Halomicronema metazoicum*, *Halomicronema excentricum*, *Halomicronema* sp. SCyano39, *Halomicronema* sp. PCyano40, *Halomicronema* sp. Goniastrea-1, *Nodosolinea nodulosa* UTEX 2910, *Oscillatoria neglecta* IAM_M-82, *Pseudanabaena constantiae*, and *Plectonema* sp. F3., whereas it exhibited about 85% pairwise sequence identity with *Gloeobacter violaceus* PCC7421 and *Pseudanabaena* PCC7403, *Synechococcus* sp. According to morphological and molecular characterizations, we ascribed this undetermined cyanobacterium strain to the *Halomicronema* genus, indicating it as *Halomicronema* sp., and assuming that it needed further analyses in order to be fully characterized. 

### 2.3. Comparisons of Toxicity Tests

**Figure 4:**
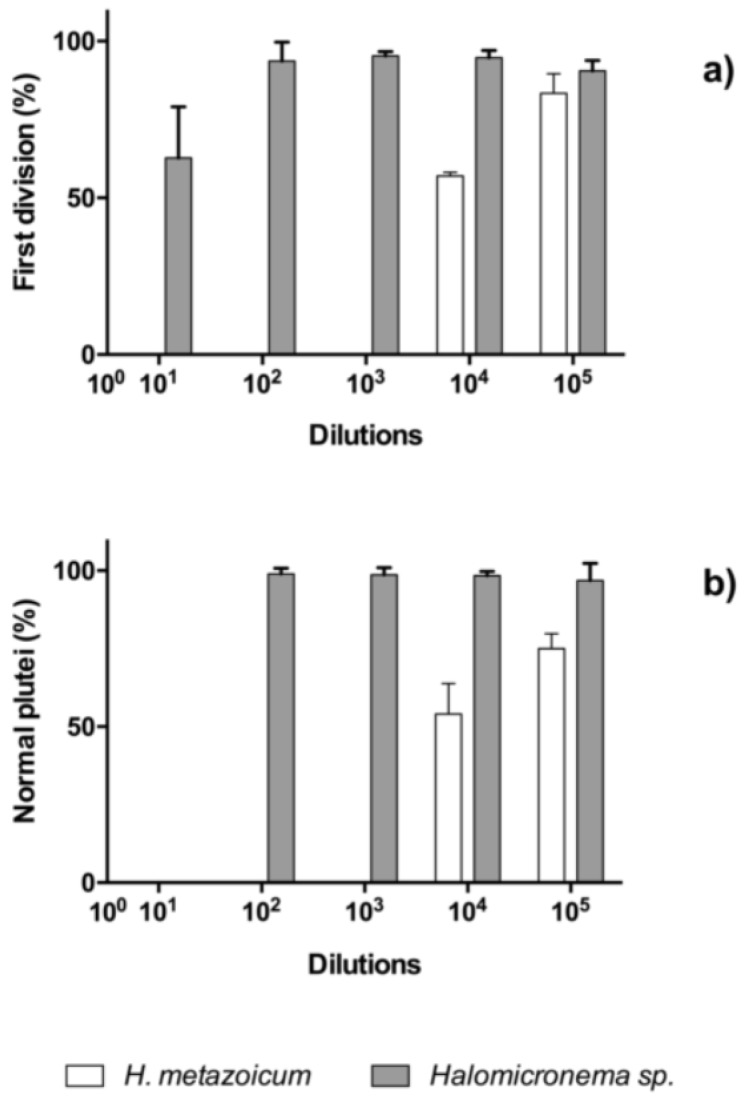
Toxicity of *Halomicronema* sp. and *H. metazoicum* spent media measured on sea urchin embryos at the first division (**a**); and as a percentage of normal plutei produced (**b**) at various dilutions of spent medium, from 1:10 to 1:100,000. The results of toxicity tests at one hour (rates of first division and percentage of normal plutei produced) were normalized over the effects on controls, to facilitate comparisons, according to the relationship (1) reported in methods. At the lowest dilutions, *H. metazoicum* triggered the block of divisions (**a**), and, consequently, a lack of normal plutei (**b**).

**Figure 5:**
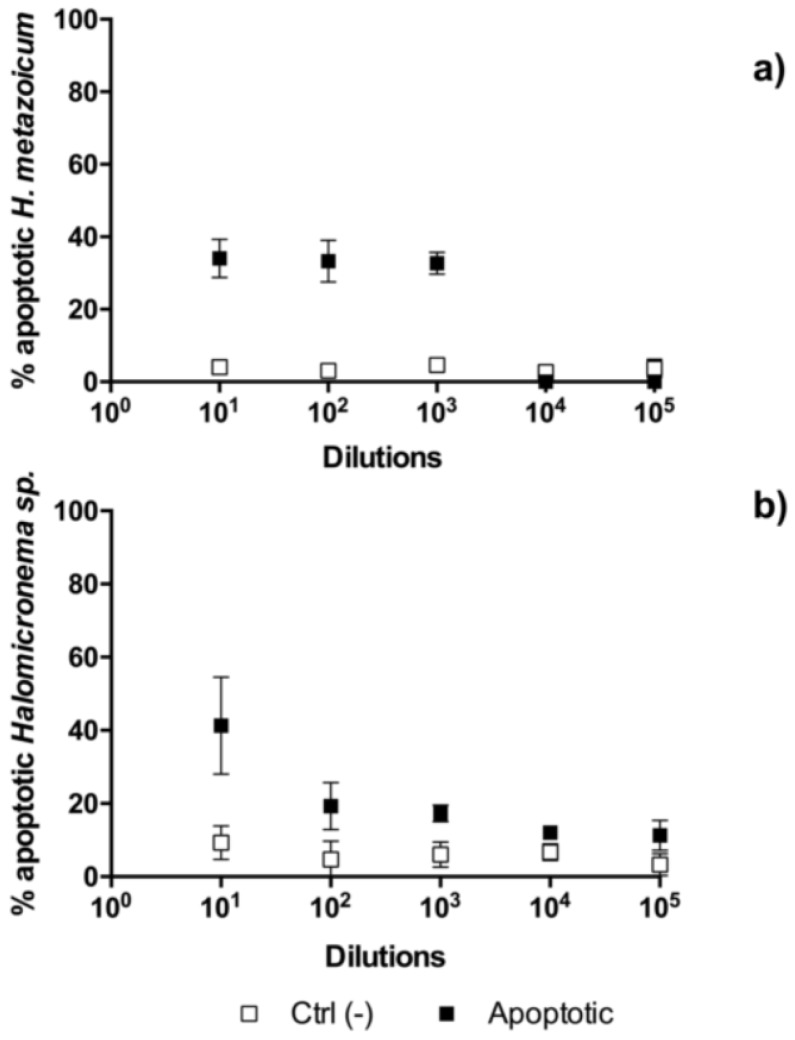
Toxicity of *Halomicronema metazoicum* (**a**) and *Halomicronema* sp. (**b**) spent medium measured on sea urchin embryos one hour after fertilization by recording the percentage of apoptotic embryos in controls and in treated samples at various dilutions of the media, from 1:10 to 1:100,000. Ctrl (−) indicates the negative control.

**Figure 6:**
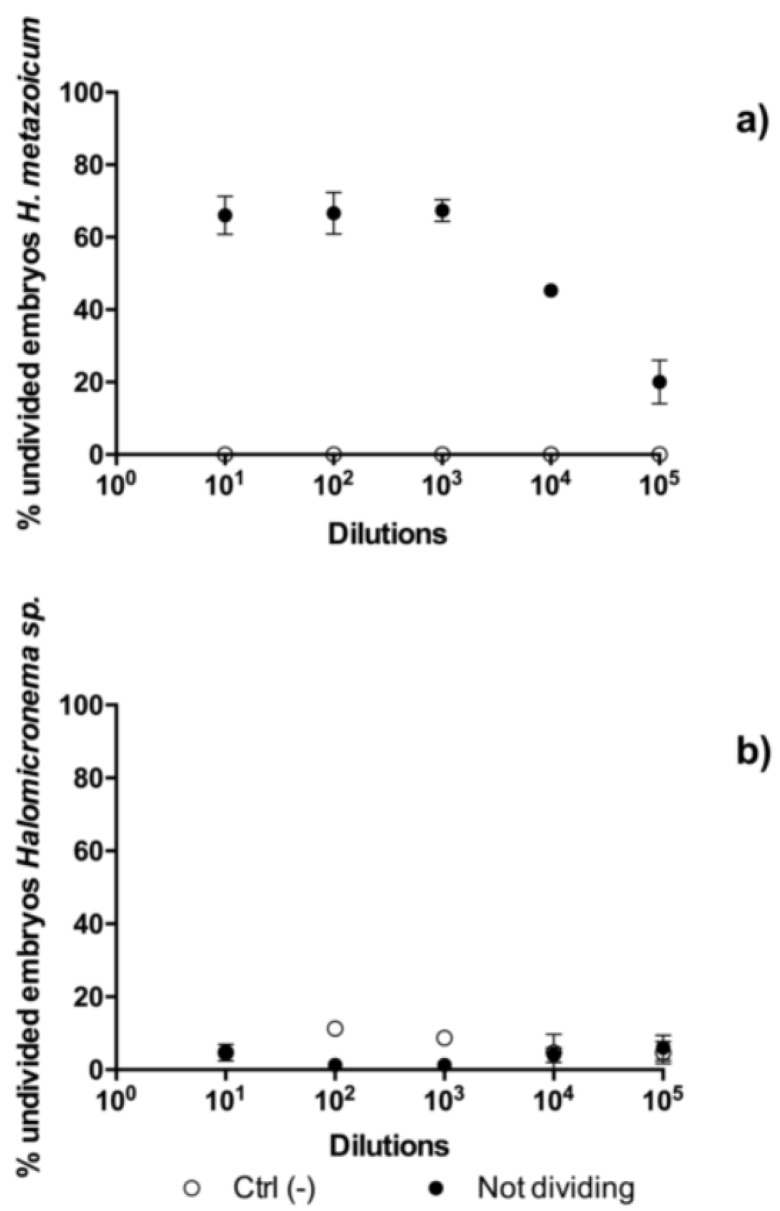
Toxicity of *Halomicronema metazoicum* (**a**) and *Halomicronema* sp. (**b**) spent medium measured on sea urchin embryos one hour after fertilization by recording the percentage of undivided embryos in controls and in treated samples at various dilutions of the media, from 1:10 to 1:100,000. Ctrl (−) indicates the negative control.

**Figure 7:**
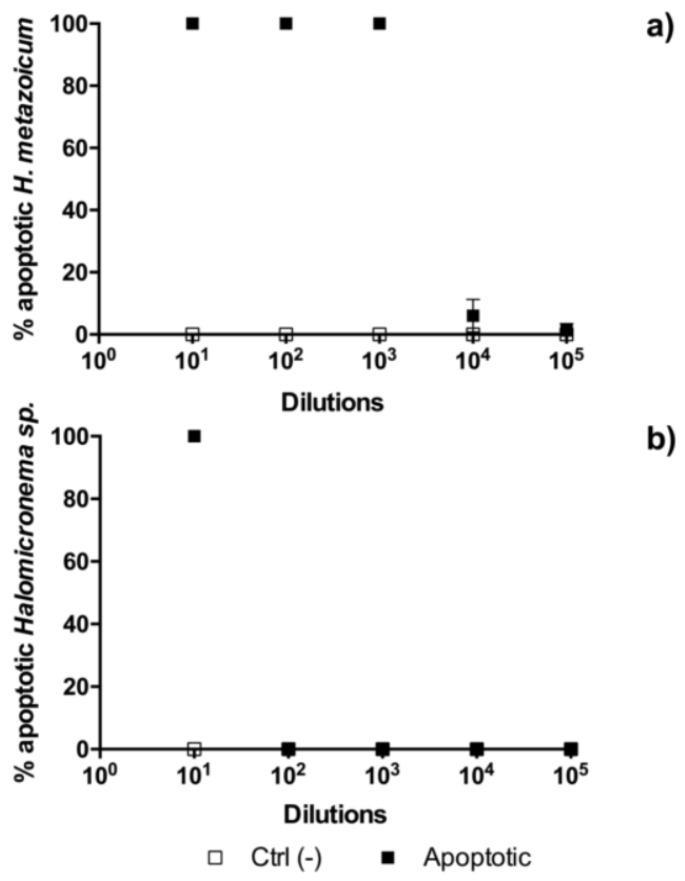
Toxicity of *Halomicronema metazoicum* (**a**) and *Halomicronema* sp. (**b**) spent medium measured on sea urchin embryos 48 h after fertilization by recording the percentage of apoptotic embryos in controls and in treated samples at various dilutions of the media, from 1:10 to 1:100,000. Ctrl (−) indicates the negative control.

**Figure 8:**
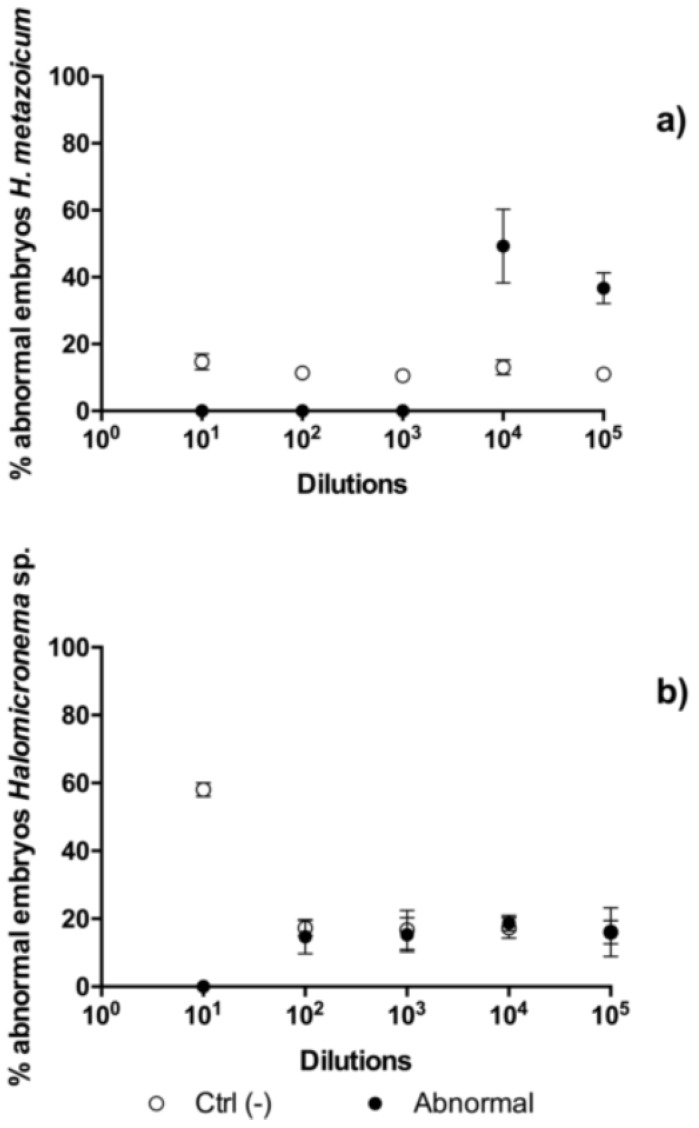
Toxicity of *Halomicronema metazoicum* (**a**) and *Halomicronema* sp. (**b**) spent medium measured on sea urchin embryos 48 h after fertilization by recording the percentage of abnormal larvae in controls and in treated samples at various dilutions of the media, from 1:10 to 1:100,000. Ctrl (−) indicates the negative control.

The results of toxicity tests one hour after the fertilization of sea urchin embryos showed clear effects of *H. metazoicum* even at low concentrations of the spent medium, up to 1/10,000 (Figure 4), both in terms of the block of the first division (Figure 4a) and the production of larvae (Figure 4b), after normalization over controls. The spent medium of *Halomicronema* sp. that was collected in thermal waters exhibited quite a lower toxicity, reducing the percentage of first divided embryos only at concentration of 1:10 (Figure 4a). At the same concentration, consistently, normal plutei were not produced (Figure 4b), but negligible effects were observed at higher dilutions. Statistical comparisons confirmed that one hour after fertilization the effects induced by the spent medium of *H. metazoicum* and those of the fresh medium were significantly different, up to a dilution of 1:10,000 (Figure 5a). At higher dilutions, no significant effects were recorded. In contrast, *Halomicronema* sp. triggered a clear increase of apoptotic embryos (i.e., embryos presenting clear blebbing formation) at the highest concentration (dilution 1:10), and very low effects at further dilutions (Figure 5b). As well, when the percentage of divided embryos in test replicates and their controls was statistically compared, we demonstrated that the spent medium of *H. metazoicum* triggers a significant effect with respect to controls, up to the highest dilutions (Figure 6a), while the effect of the spent medium of *Halomicronema* sp. is negligible at any concentration (Figure 6b). An effect of the control medium was recorded, because the BG11 medium is quite dense, and it impacts the physiology of embryos at the highest concentrations (Figure 6b). These results are mirrored by the records obtained 48 h after fertilization. In fact, *H. metazoicum* triggered the production of apoptotic embryos at all concentrations, with a clear threshold above a dilution of 1:10,000 (Figure 7a), while *Halomicronema* sp. had an effect only at the highest concentration, with respect to controls (Figure 7b). No effect of the control medium was recorded on apoptotic processes, even at the highest concentrations. The effect of spent medium on the production of abnormal plutei was less evident. However, the remaining percentage of viable embryos (that were still present at dilutions higher than 1:1000, because they were not impacted by the apoptotic processes reported in Figure 7a) exhibited malformations in the replicates treated with *H. metazoicum* spent medium (Figure 8a); in contrast, a clear effect was produced by the BG11 medium that was used to culture *Halomicronema* sp. at the highest concentration (Figure 8b), but no effects of the spent medium were detected at any concentration.

**Figure 9:**
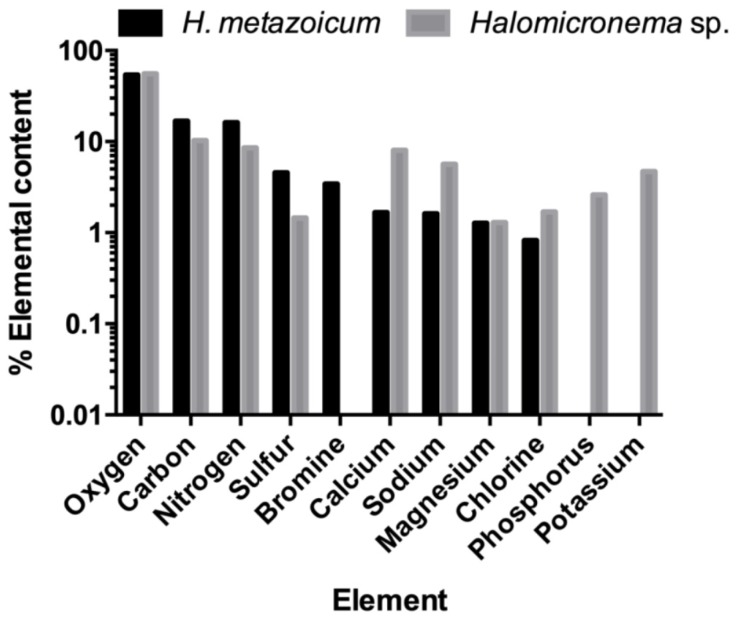
Elemental composition of *Halomicronema* sp. and *H. metazoicum* measured by means of X-ray diffraction using a high-performance SEM Phenom ProX.

These effects are due to compounds constitutionally produced and excreted in the spent medium, but the cells of bacteria are characterized by clear differences in composition (Figure 9) detected by the high-performance scanning electron microscope (SEM). In fact, X-ray analyses detected similar contents of constitutive elements such as oxygen, carbon, and nitrogen, but *H. metazoicum* actively stored larger amounts of bromine and sulfur, while *Halomicronema* sp. was characterized by higher concentrations of calcium, sodium, and chlorine. As well, potassium and phosphorus were detected exclusively and in large amounts in the species collected in thermal waters.

**Figure 10:**
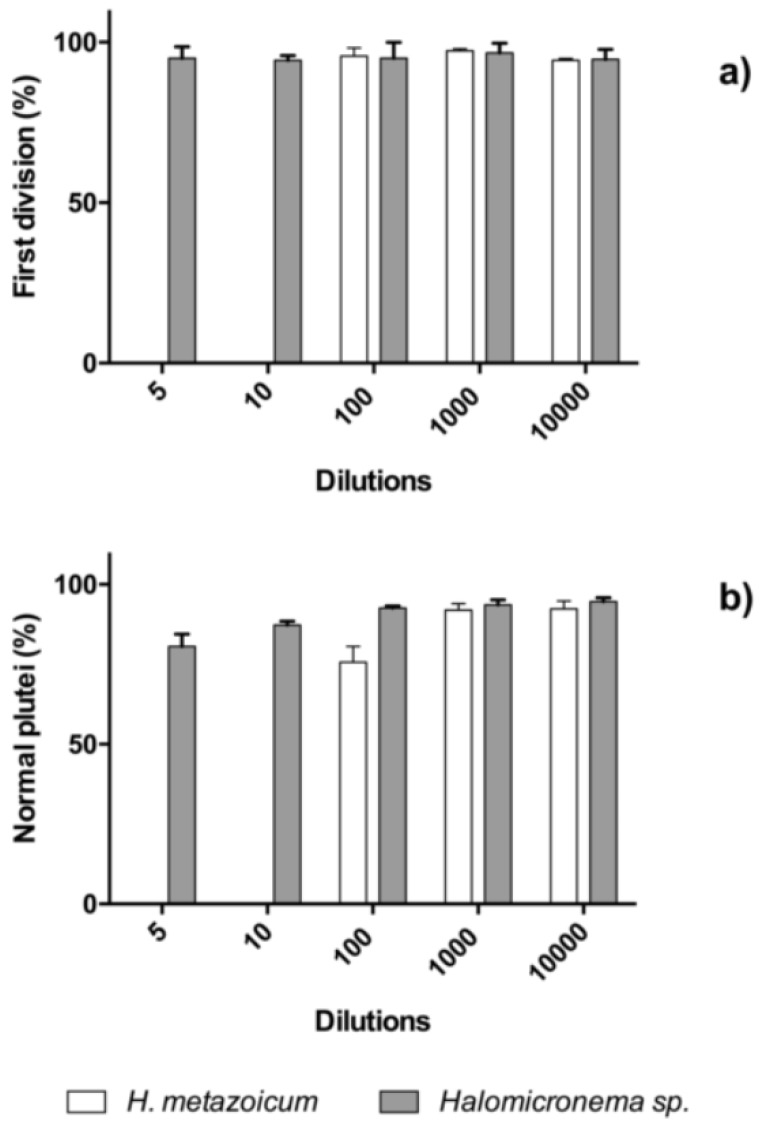
Toxicity of *Halomicronema* sp. and *H. metazoicum* cell homogenates measured on sea urchin embryos at the first division (**a**), and as a percentage of normal plutei produced (**b**) at various dilutions of spent medium, from 1:5 to 1:10,000. The results of toxicity tests at one hour (rates of first division and percentage of normal plutei produced) were normalized over the effects on controls, to facilitate comparisons, according to the relationship (1) reported in methods. At the lowest dilutions, *H. metazoicum* triggered an absence of divisions (**a**), and consequently, a lack of normal plutei (**b**).

The toxicity tests that were performed on homogenates of bacterial cells confirmed a low toxicity of *H. metazoicum,* which was exhibited at the highest concentrations (1:5–1:10), while lower concentrations were innocuous for sea urchin embryos at the first division, and triggered a moderate effect on plutei development after 48 hours (Figure 10). In contrast, *Halomicronema* sp. cells exhibited an absence of toxigenic activities both at the first division and during the development of plutei. A very low activity was exhibited only at the highest concentrations after 48 hours, which is comparable to the one produced by their culture medium (Figure 10b). ANOVA tests indicated that all of the described differences are significant at *p* < 0.001.

## 3. Discussion

The two species of cyanobacteria recently isolated are characterized by distinct morphological, ultramorphological, and chemical properties. They are both thin filamentous cyanobacteria, but TEM analyses indicate peculiar cellular features and different trends in the mat aggregations. *Halomicronema* sp. needed a richer culture medium and tended to remain in the water column, while *H. metazoicum* demonstrated maximum vitality in *f/2* and a tendency to settle to produce growing mats. This is in line with the environmental conditions of the collection sites, since the latter was found epiphytic on seagrass leaves, and the first was collected dispersed in the thermal waters. The thin filamentous genera of cyanobacteria such as *Oscillatoria spp*, *Phormidium spp,* and *Plectonema* spp. are known to be polyphyletic [4,11,43]; this probably increases their large physiologic plasticity and genetic diversity, favoring the possibility of colonizing extreme environments. Our polyphasic investigation indicates that the two species herein considered are different, and the undetermined one will need accurate analyses to be characterized, since the 16S rRNA molecular signatures failed at finding a clear determination based on comparisons with known taxa. In fact, as forecasted by previous authors [35,49], “this genus should be further extended in the future, to include more isolates from hypersaline environments”, which are known to be elective habitats for thin filamentous non-heterocystous cyanobacterial populations. 

Toxicological analyses added some important details, since the species living in thermal waters, which were characterized by low competition levels with other organisms and adapted to extreme conditions of salinity and temperature [49], exhibited the lowest toxicity, as constitutionally expressed both in their culture medium and in the cell homogenates. In contrast, *H. metazoicum* living as an epiphyte of seagrass leaves is subjected to a strong competition for space and nutrients prompted by a complex association of algae and animals, and it elicited, indeed, the biosynthesis of an effective set of chemical weapons. There are remarkable effects detected by standard toxicity tests on sea urchin embryos [50] that demonstrate that this species is able to keep its area of proliferation clear of competing species of epiphytes. As well, the toxicity contained in their homogenates may represent an efficient defence against possible consumers as various grazers living in the seagrass leaf stratum. It is also interesting to consider that the same species was firstly set as an endobiont of marine metazoans [42], and it will be worth investigating whether any difference in the production of toxic metabolites exists between the two strains of *H. metazoicum*, since such a toxigenic power could be in contrast to a physiologic cooperation with hosts. Previous investigation [48] indicated that specific alkaloids, such as cylindrospermopsins (CYN), are synthesized by selected cyanobacteria, and they are involved in allelopathic responses, providing an advantage over sympatric species in habitats characterized by complex antagonistic relationships. In addition, the levels of production of allelopathic compounds may be modulated by *Microcystis aeruginosa* according to the needs imposed by the presence of competing species and their actual density in the medium. These relationships produce a dense network of chemical communications that is yet to be fully understood, but it is evident that two specific features (namely, the ability to colonize extreme environments and the secretion of several chemical weapons) explain the evolutionary potential of cyanobacteria in various geographical regions.

In our case, the toxigenic power of *H. metazoicum* was demonstrated at various levels, inducing fertilization failure (personal observation), antimitotic power, apoptogenic power, and mutagenic influence. Thus, various compounds might be contemporaneously produced and liberated in the culture medium, to control the presence of assorted categories of competitors. Alternatively, the same compound could have different modes of action depending on its initial levels of production, as demonstrated in other species [48]. This complex set of chemical weapons will be further investigated, since extracellular compounds could be used for biotechnological purposes (e.g., for medical and anti-parasitic purposes), given the marked biological activity here demonstrated. In contrast, *Halomicronema* sp. spent medium exhibited a very low antimitotic power, only at the highest concentrations, even after longer exposure times. This apparent lack of allelochemical power fits with the features of the extreme environment where it was isolated, because target competitors and possible consumers are virtually absent. However, the induction of apoptosis has been demonstrated, and this could lead in the future to therapeutic applications, since it is associated with low generic toxicity levels. These features indicate the different possible applications for the compounds constitutively secreted by the two species: strong toxigenic activity against metazoans for *H. metazoicum*, even at low concentrations, and specific apoptogenic activity that is potentially useful for medical applications in the case of *Halomicronema* sp. Further tests and chemical characterizations will explore these hypotheses.

The elemental composition of the two species that were obtained by taking advantage of the high-performance SEM analyses confirmed these differences [51]. The larger amounts of phosphorus and potassium in *Halomicronema* sp. could be a consequence of the different culture media needed for the two species, since some compounds that were abundant in the medium were certainly stored in the cells. More interesting is the difference among the sulfur, bromine, and chlorine concentrations. In fact, brominated organic compounds are known to be produced during cyanobacteria blooms [52], and the substitution of Cl^−^ with Br^−^ is a typical process occurring during the biosynthesis of these compounds [53]. For example, dibromoacetic acid produced by freshwater cyanobacteria is known to be toxic for nematodes, and even for mammalians [54]. Since the difference in the contents of these elements is specular in the two species, we could hypothesize a different efficiency in the pathways of biosynthesis, leading to dissimilarities in their toxigenic power, and fitting their adaptations to distinct environments.

## 4. Conclusions

Evidently, the chemical defences of *Halomicronema* spp. are tuned according to local needs, and the data herein collected indicate that even their alternative presence in benthic or planktonic environments could be characterized by differences in the chemical ecology of their populations. Thin cyanobacteria are definitely demonstrated to be quite strong organisms, controlling the populations of their competitors and possible consumers priming antimitotic, apoptogenic, and teratogenic effects, but their constitutive production and secretion in the surroundings is evolved according to environmental constraints. For example, colonial cyanobacterial blooms are responsible for the deterioration of freshwater systems with increasing frequency and intensity, also due to global warming [31,55], and this hardly impacts the possible uses of those water masses for humans. In addition, a conserved phylogenetic structure was observed in the cyanobacterial populations for several lake samples [28], confirming our assumption that phylogenetic traits are paired with the ability of cyanobacteria to settle in given environmental conditions. Thus, phylogenetic and evolutionary constraints are paired with physiologic traits and toxigenic properties of cyanobacteria, directing their adaptability to a wide range of environments, including extreme physical conditions. This evidence may on one side help the comprehension of their patterns of geographical distribution and expansion; on the other side, it may direct our attention toward strains characterized by peculiar biological activities, in the search of active compounds for interesting biotechnological applications.

## 5. Materials and Methods 

### 5.1. Collections of Cyanobacteria in Seawater

Leaves of *Posidonia oceanica* were collected in April 2017 by scuba divers, at five meters depth, in a meadow off Lacco Ameno d’Ischia (Bay of Napoli, Italy: 40°44′56″ N, 13°53′13″ E). At the collection site, the temperature was about 19 °C, pH 8.12, salinity 38 psu, at an irradiance of about 400 µE m^−2^. After their arrival in the laboratory, they were reared in aerated closed-cycle tanks containing sterilized water. An inspection under a stereomicroscope permitted the detection of small portions of various epiphytes, which were promptly collected by forceps and moved into multi-well plates with Guillard’s *f/2* medium (Sigma Aldrich, Milan, Italy). Their development was recorded while they were kept in a thermostatic chamber at 18 °C, with an irradiance of 200 µE m^−2^, and a 12:12 hour dark/light cycle. When axenic thalli were obtained, they were further transferred every two to three days in multi-wells containing fresh *f/2* medium, up to a complete isolation. The first identification of the strains was performed under a light microscope, and was based on the filament and cell shape and size, the shape of the terminal cell, the presence of calyptra, sheaths, and the type of reproduction. 

### 5.2. Collection of Cyanobacteria in Thermal Waters

A surface water sample rich in algal mats was collected in sterile bottles in June 2012 from a thermal pond near Citara beach, Ischia Island, Italy (13°51′45″, 40°43′12″) and transported in the microalgae lab of the CAISIAL Centre (Portici, Napoli) at about 15 °C in dark. The temperature of water, which is classified as sulfate–chloride–alkaline, during sampling was 36.8 °C with a pH of 8.5 and salinity of 39.6 psu. The cyanobaterial strain was isolated from other microorganisms by the repeated transfer of isolated trichomes on agar plates of BG11 medium [46] in artificial seawater (salinity 38 psu, pH 8.5). After isolation, a culture was established in a thermostastic chamber at 25 ± 1 °C, with a light intensity of 200 µE m^−2^ and a 12:12 h dark/light cycle. A living culture of the cyanobacteria strain has been deposited at the Algal Collection (ACUF) at the Biology Department of Naples University Federico II (Italy). 

### 5.3. Light and Electron Microscopy

The identification and morphological characterization of cyanobacteria were performed by light microscopy (Nikon Eclipse E-800). Samples for TEM were fixed with 1% glutaraldehyde in sodium cacodylate buffer and post-fixed with 1% osmium tetroxide in the same buffer, dehydrated in a graded alcohol series, embedded in epoxy resin Epon 812, and sectioned at 650 nm with an Ultracut. The sections were stained with 1% Uranyl Acetate Replacement Stain “UAR” (EMS Science LTD) and lead citrate, and examined under TEM operating at 100 kV. 

In addition, we took advantage of a new Phenom ProX desktop SEM (ThermoFisher Scientific, Waltham, MA, USA) to obtain high-resolution images of both species along with elemental analyses. This microscope permits, alongside acquiring high-resolution images, identifying the different chemical elements present on a specimen and we used it to with an EID (identification software package specifically designed and fully integrated with an energy dispersive spectrophotometer) to detect any difference in the elemental composition between the two species.

### 5.4. Molecular Identification

Individual cell cultures of both strands were collected and concentrated by centrifugation for 20 min at 4500 rpm (revolution per minute) at 4 °C, and then frozen at −20 °C until use. About one gram of DNA has been used for DNA extraction according a method reported in Ruocco et al. [29] for *H. metazoicum*, and previously described by Singh et al. [56]. Further, the genomic DNA concentration was quantified by NanoDrop spectrophotometer (ND-1000 UV–vis Spectrophotometer; NanoDrop Technologies, Wilmington, DE, USA) and its integrity was evaluated by electrophoresis on 0.8% agarose gel. The gene coding for a 16S rRNA subunit was amplified by polymerase chain reaction (PCR) using five cyanobacteria-specific universal primer pairs: 1) forward primer CYA106F with an equimolar mixture of reverse primers CYA781R(a) and CYA781R(b) [57], amplifying a fragment of 600 bp; 2) forward primer CYA783F with reverse primer S17 [58], amplifying a 600-bp fragment; 3) forward primer P1 with reverse primer P2 [59], amplifying a 1200-bp fragment; 4) forward primer pA with reverse primer B23S [60], amplifying a 1300-bp fragment; and 5) forward primer CYA359F with an equimolar mixture of reverse primers CYA781R(a) and CYA781R(b) [57], amplifying a fragment of 600 bp. PCR products were subjected to the DNA sequencing of both strands. The total 16S region was aligned to Gene Bank using the Basic Local Alignment Search Tool (BLAST [61]) and then aligned with highly similar sequence using MultiAlin (http://multalin.toulouse.inra.fr/multalin/). For phylogenetic analysis, 14 16S rRNA gene sequences of cyanobacteria (including *Cyano_Pos* and *Halomicronema* sp.) have been considered (see Table S1 for species’ names and accession numbers). Multiple sequence alignments were conducted using the CLUSTALW program (http://www.genome.jp/tools-bin/clustalw). Multiple sequence alignments were performed using the service named FASMA, which is available at http://bioinformatica.isa.cnr.it/FASMA/ ([62]; Table S2). 

### 5.5. Toxicity Tests on Media

Both strains of cyanobacteria were subjected to standard toxicity tests [63] in order to compare the toxigenic activity of their spent media using an established model: the embryos of the sea urchin *Paracentrotus lividus*. To this end, cyanobacteria were cultivated in the above-mentioned conditions for one month, in order to facilitate the accumulation of secondary metabolites in the medium. The spent media were collected at the end of the production period, filtered over a 0.22-µm Millipore filter, stored in glass vessels, and kept at −20 °C up to the start of bioassays. Prior to performing the tests, the contents of two replicate samples produced by each species were pooled. The medium was sampled and diluted at various concentrations in filtered seawater for both tested species. Basically, we took into account the following dilutions of the culture medium in filtered seawater: 1:10, 1:100, 1:1000, 1:10,000, and 1:100,000 in volume. Bioassays were prepared starting from two mature females and two males of *P. lividus* collected in the Bay of Napoli. They were injected with 1 mL of 0.5 M KCl into the coelom through the soft derma around the mouthparts, in order to stimulate the contraction of gonads. The sea urchins were vigorously shaken, and females were placed with their mouths up, over a 50 mL beaker, until the gametes were released into filtered (0.22 µm Millipore, Billerica, MA, USA) seawater to facilitate the collection of oocytes, which were rinsed three times with clean seawater to remove possible organic residuals [64]. Sperms were collected “dry”, after the injection of 1 mL of 0.5 M KCl into the coelom of males, using a Pasteur pipette and sucking over the surface of gonopores, to avoid premature activation. The gametes obtained from each individual were conserved in plastic vessels up to fertilization. Sub-samples of oocytes were collected and added with a drop of sperm suspension. Normal egg activation was revealed by the elevation of the fertilization membrane within 40–80 s, appearing as a clear circle. Pools of embryos exhibiting percentages of fertilization lower than 95% were discarded. Pools exhibiting viable embryos were used for bioassays. To this end, embryos produced by the two females were pooled, and 400 embryos were collected in triplicate and transferred into multi-well dishes filled with four mL of the appropriate dilutions of cyanobacteria culture media, as above specified. Controls were prepared using only seawater added with corresponding proportions of freshly prepared culture medium. The results were recorded at various time intervals and according to each concentration. In particular, after one hour, the multi-wells were observed under a Leica Z6Apo macroscope to record the percentage of individuals showing normal cell division and, eventually, hallmarks of apoptosis (detected by recording nuclear and cellular condensation and fragmentation under optical microscopy), some of which were interesting. After 48 h, the contents of the wells were fixed with the addition of a drop of 4% buffered formalin and examined to record the percentage of normal plutei, abnormal plutei, or apoptotic embryos.

### 5.6. Toxicity Tests on Homogenates of Bacterial Cells

Bacterial cells were collected from live cultures, centrifuged, and weighed. Four replicates (100 mg each) of fresh cyanobacteria were fragmented into a ceramic mortar, and then transferred into a chilled tube and sonicated twice for one minute, up to a complete homogenization of citoplasmatic contents. The resulting suspension was centrifuged for 10 min (1062 G), and the supernatant was collected, filtered over a 0.22 µm Millipore membrane, brought to a volume of 5 mL with the addition of filtered and sterilized seawater, and then frozen up to the dilutions for bioassays. The supernatant was melted and further diluted in sterilized seawater at concentrations of 1:5, 1:10, 1:100, 1:1000, and 1:10,000 to test the toxicity of cyanobacterial cell contents as compared to their spent media, as above indicated.

### 5.7. Statistical Analyses

Data were organized as means and their standard deviations from various replicates, for each set of measurements. The results of toxicity tests at one hour (rates of first division and percentage of normal plutei produced) were normalized over the effects on controls, in order to facilitate comparisons, according to the relationship:

(1) $$\%\;\mathrm{normal}\;=\;\frac{\mathrm{nr}.\mathrm{normal}\;\mathrm{larvae}\;\mathrm{in}\;\mathrm{test}\;\mathrm{replicates}}{\mathrm{nr}.\mathrm{normal}\;\mathrm{larvae}\;\mathrm{in}\;\mathrm{controls}}\times 100$$

where % normal is the rate of first division observed in each replicate and their controls, or the percentage of normal plutei observed in replicates and their controls.

All of the datasets on the toxigenic effects, i.e., the percentage of embryos undergoing the first division in test vessels versus controls, the percentage of normal plutei counted one hour after the fertilization, the percentage of apoptotic embryos recorded one hour and 48 h after fertilization, as well as percentage of not-divided embryos recorded (one hour and 48 h after fertilization) were analyzed using one-way and two-way ANOVA, after checking the homogeneity of variance, and Sidak post-hoc tests were applied to check the contribution of each factor to the differences that were observed. Data were tested for normality and homogeneity of variances by the D’Agostino–Pearson and Levene’s tests, respectively. Graphs and statistical analyses were computed using the software GraphPad Prism version 7.00 for Macintosh (GraphPad Software, La Jolla, CA, USA, www.graphpad.com) and Statistica version 10 (StatSoft Inc., Tulsa, OK, USA).

## Supplementary Materials

The following are available online at http://www.mdpi.com/2072-6651/11/2/99/s1. Table S1: Specie names (including *Cyano_Pos* and *Halomicronema* sp., analyzed in the present work), acronyms (used in the phylogenetic tree of Figure 3) and accession numbers of cyanobacteria used for phylogenetic analysis of 16S rRNA gene sequences. Table S2: Sequence identity percentage of the fourteen cyanobacteria species used for phylogenetic tree.
