# Supplementary material for: Distribution of Toxigenic Halomicronema spp. in Adjacent Environments on the Island of Ischia: Comparison of Strains from Thermal Waters and Free Living in Posidonia Oceanica Meadows
Source: Toxins (Basel). 2019 Feb 8;11(2):99. doi: 10.3390/toxins11020099 (PMC6409854; doi:10.3390/toxins11020099)
Supplement: Supplementary file 1 [file toxins-11-00099-s001.zip › Table_S1 for final.docx]

Supplementary Materials: Distribution of Toxigenic *Halomicronema* spp. in Adjacent Environments on the Island of Ischia: Comparison of Strains from Thermal Waters and Free Living in *Posidonia Oceanica* Meadows

Valerio Zupo, Mirko Mutalipassi, Nadia Ruocco, Francesca Glaviano, Antonino Pollio,
Antonio Luca Langellotti, Giovanna Romano and Maria Costantini

**Table 1.** Specie names (including *Cyano_Pos* and *Halomicronema* sp., analyzed in the present work), acronyms (used in the phylogenetic tree of Figure 3) and accession numbers of cyanobacteria used for phylogenetic analysis of 16S rRNA gene sequences.

| **Name** | **Acronym** | **Accession number** |
| --- | --- | --- |
| *Cyanobacterium_* | *Cyano_Pos* |  |
| *Gloeobacter violaceus PCC 7421* | *G. violaceus PCC 7421* | AF132790.1 |
| *Halomicronema sp.* |  |  |
| *Halomicronema sp. SCyano39* | *H. sp. SCyano39* | DQ058860.1 |
| *Halomicronema sp. PCyano40* | *H. sp. PCyano40* | DQ058890.1 |
| *Halomicronema excentricum TFEP1* | *H. excentricum* | AF320093.1 |
| *Halomicronema sp. Goniastrea-1* | *H. sp. Goniastrea-1* | AB257773.1 |
| *Halomicronema metazoicum ITAC101* | *H. metazoicum ITAC101* | GU220365.1 |
| *Nodosolinea nodulosa UTEX 2910* | *N. nodulosa UTEX 2910* | EF122600.1 |
| *Oscillatoria neglecta IAM M-82* | *O. neglecta IAM M-82* | AB003168.1 |
| *Plectonema sp. F3* | *Plectonema sp. F3* | AF091110.1 |
| *Pseudanabaena PCC7403* | *Pseudanabaena PCC7403* | AB039019.1 |
| *Pseudanabaena constantiae* | *P. constantiae* | DQ393595.1 |
| *Synechococcus sp.* | *Synechococcus sp.* | D88288.1 |
